# Cross-Regional Research in Demographic Impact on Safety Consciousness and Safety Citizenship Behavior of Construction Workers: A Comparative Study between Mainland China and Hong Kong

**DOI:** 10.3390/ijerph191912799

**Published:** 2022-10-06

**Authors:** Xiangcheng Meng, Alan H. S. Chan

**Affiliations:** 1 Sub-Institute of Public Security, China National Institute of Standardization, Beijing 100191, China; 2 Department of Advanced Design and Systems Engineering, City University of Hong Kong, Hong Kong 999077, China

**Keywords:** comparative study, safety consciousness, demographic influence, cross-regional analysis, safety citizenship behavior

## Abstract

The construction industry has rapidly developed with continuous prosperity in Hong Kong and Mainland China, although accidents still occur with unacceptable frequency and severity. For promoting the safety issue of workers in construction industry, safety citizenship behavior (SCB) and safety consciousness (SC) were considered two influential constructs and further studied with integration of sociodemographic theories by scholars. However, no study has compared the SC and SCB of construction workers in terms of the demographic influence between Mainland China and Hong Kong. To fill this research gap, this study investigated the territorial difference between these two regions by conducting a cross-sectional questionnaire survey with recruitment of 253 Mainland construction workers and 256 Hong Kong construction workers. Significant similarities and differences of SC and SCB performance were revealed in terms of the workers with different genders, education levels, weekly working hours, and ages. This study provides insights into the comparison of demographic influence on SC and SCB of construction workers between Hong Kong and Mainland China, which is unique as it can yield useful managerial knowledge relevant to the personal safety of targeted groups of construction workers with particular demographic characteristics in both regions and contribute the implementation of safety interventions in line with the specific distinction in the territorial aspect.

## 1. Introduction

Construction safety in China has become a primary concern for people due to the tremendous losses caused by work-related injuries of personnel [1,2]. The National Bureau of Statistics of China reported 5.21 injuries for every 100 workers and 14.31 fatalities for every 100,000 workers in Mainland China’s construction industry in 2018 [3]. Similarly, high fatality rate of construction workers was observed in Hong Kong, which is the special administrative region of China. For example, 3.17 injuries were reported for every 100 workers and 12.51 fatalities were observed per 100,000 workers in 2018 [4]. These statistics highlight the urgent need for all personnel and academics concerned with occupational health and safety in both Mainland China and Hong Kong to reduce the number of accidents. To address this issue, scholars have considered safety citizenship behavior (SCB) and safety consciousness (SC) as noteworthy topics for investigation of personnel safety [5,6,7]. As defined, SC is considered a kind of mental perception and understanding of environmental safety and working circumstance of individuals, which is highly related to the improvement of behavioral safety levels and humanity living environments [8]. SCB can be interpreted as a voluntary behavior focusing on improving the organizational safety performance and mutual support at group level among employees [9], which is usually shown as mutual voluntary assistance among working members to achieve safety improvement [5]. Meng et al. further expanded the research of SC and SCB theories by developing the scale measurements [10]. However, the research gap regarding the missing comparison of SC and SCB between two regions hindered the implementation of safety interventions related to SC and SCB that target specific groups of construction workers in these two regions. Considering the regional background, the subtle administrative relationship and similar developing stages of societies and economies were observed with regard to the political and cultural aspects. Hong Kong, as one of the special administrative regions of China, identifies traditional Chinese culture within an abstract and detached sense while its administrative mode is distinguished with a typical communist regime in Mainland China. For the social development aspect, the Hong Kong government has maintained a capitalist system, and it has incorporated local and overseas elites into administration and political consultations because of its colonial background, which previously situated Hong Kong at a leading position.

The regional similarity and difference may further lead to influential distinction of occupational safety of the construction personnel under the specific circumstance [7]. However, the seriousness of fatal accidents in the construction industry was still reported in both regions with no research comparing or discovering the possible reasons in terms of specific characteristics of the construction personnel. To fill such gap, this paper aims to identify and compare the difference in SC and SCB between Mainland China and Hong Kong, including the influence of demographic factors, such as gender, education level, age, and weekly working hours, since the previous studies have demonstrated the remarkable influence of personal characteristics (gender, age, educational background, etc.) on personnel safety [8,11,12]. This paper provides a starting point for further research on the comparative study of demographic influences on the personnel safety of construction workers between regions. It then analyzes the reasons for and the resultant implications of the territorial differences, which can help these regions learn complementarily from one another.

## 2. Materials and Methods

### 2.1. Theories of SC and SCB

SC refers to the positive individual attitude and perception of environmental safety and working circumstances [13]. The safety behavior of humans and their living environment can be enhanced by promoting safety consciousness [6,7]. SCB is an organization-based voluntary behavior that focuses on improvement of mutual support and safety performance at the organizational level [9]. Compared with traditional safety behavior, which focuses on the individual acts for ensuring personal safety, SCB is considered preferable for safety research because it is more related to the overall safety of the organization [5]. Meng et al. developed scales to measure SC and SCB for the construction industry, which were also applied in the present study due to the considerable reliability and validity [10].

Comparing the safety constructs between these two regions is deemed feasible and valuable given the subtle relationship between Mainland China and Hong Kong. Specifically, Hong Kong functions within a legal environment with a capitalistic state but still partially adheres to traditional Chinese cultural patterns [14,15]. This study compares the SC and SCB between Mainland China and Hong Kong construction workers given that the safety of construction personnel is considered an important issue in both regions with different social and political conditions. For the former, personal unsafe behavior has been identified as the main cause of the high accident rates in its construction industry [16,17]. Accordingly, construction companies in Hong Kong have extensively implemented preventive measures, such as prevention-behavior-based safety management and supervisor-focused behavior-based safety methods, to improve the safety of their workers [18,19]. Meanwhile, the government of Mainland China has organized vocational education training to enhance the qualifications of construction workers in both theoretical and practical aspects [20].

### 2.2. Demographic Influence

Previous studies revealed several potential demographic variables that may cause effect towards an individual’s perception, personality, performance, and organization, and between-member interaction, including gender, age, working hours and education level [21,22,23,24,25,26,27,28]. In detail, females have been involved in fewer accidents and have a higher risk-avoiding consciousness than males [29]. Nevertheless, some discussions persist about regional ambiguity as female workers sometimes suffer higher risk due to higher job stress and injury rate than male workers [30]. Education has been considered a direct and effective strategy for improving work capability and safety skills [31], but some uncertainties regarding the regional influence of education level on the personal awareness and organizational safety of construction workers remain unaddressed due to the observed insignificant correlations [32]. For age, Schwatka and Rosecrance indicated that young workers will have better safety at work compared with older workers because of their better alertness to potential threats and higher physical capacities [33]. However, some studies have reported the opposite conclusion regarding the regional age effect on safety of workers due to the finding of preferable personnel safety of elder workers with better safety experience [34,35]. Moreover, Lee and Lee pointed out the possibility of working-hour reduction to save workers from unsafe situations [36]. Skogstad et al. found that unreasonable working hours strongly increase the likelihood of increased occupational injuries and illnesses [37], which will also cause improper work shift that causes disorders in workers’ lives and leads to health problems [38,39]. From the above, although the previous studies have proven that demographic factors significantly influence the safety of personnel, the effect will be influenced as the change of certain circumstances (such as location, culture and social background, etc.), sometimes even convert the demographic influence from positive to negative [40,41]. Therefore, to fill this research gap, the present study aims to conduct a cross-regional comparison between Hong Kong and Mainland China for the influence of demographic impact on SC and SCB, which are representative of the personnel safety of construction workers.

### 2.3. Hypotheses

Table 1 lists 17 hypotheses to clarify the cross-regional differences in the demographic effects on SC and SCB. The SC and SCB of Hong Kong workers were assumed to be higher than that of Mainland workers due to the higher level of education and technology acceptance during the rapid city development in recent years [42]. The trend of demographic influence in Hong Kong was hypothesized to be identical with Mainland China due to their similar cultural background of East Asia [15]. Moreover, the regional comparison of the intensity of the demographic effect was included in the hypotheses.

### 2.4. Methodology

#### 2.4.1. Questionnaire Survey

A cross-sectional survey was conducted on site with the assistance of local worker unions and companies from September 1st 2021 to April 30th 2022, by which the demographic information and SC and SCB data of the workers were collected. A total of 530 respondents were recruited from relevant construction enterprises (265 from Mainland China, and 265 from Hong Kong) to fill out the questionnaire, and 509 valid data were finally obtained (253 from Mainland China and 256 from Hong Kong). The questionnaire adopted in this study was previously developed and published by Meng et al. [10] for measuring the correlation between SC and SCB and is now widely applied in relevant studies due to its considerable reliability and validity [43,44]. The present study further expanded the research to the cross regional comparison between Hong Kong and Mainland China about the demographic influence on SC and SCB of construction workers. The questionnaire was translated into Mandarin and Cantonese and some descriptions of the items were revised (names of applied construction regulations, safety terms, etc.) in line with the linguistic customs and local background of Mainland and Hong Kong workers, respectively. The questionnaire included 11 SC items and 12 SCB items, which were divided into four parts in line with the dimensions of SC and SCB. The SC dimensions involved conscientiousness, familiarity with safety regulations, dependency of work experience, and education on safety skills, while the SCB dimensions included self-control, relationship between superiors and subordinates, mutual aid, and participation in suggestion making. All items were rated on a five-point Likert scale ranging from 1 (“highly disagree”) to 5 (“highly agree”). Before answering the questions, the respondents were asked for their demographic information, including their education level, age, gender, and weekly working hours. For research ethics, all respondents granted their informed consent by signing their names before answering the questionnaire.

All participants provided written (or electronically displayed) informed consent before participating in this study and agreed to the terms and conditions. This study did not require the participants to be involved in any physical and/or mental intervention. Participants’ information was anonymized, and deidentified prior to analysis. This research did not obtain identifiable private information. This research gained the approval of the Ethics Sub-committee of Research Committee in City University of Hong Kong (approval number: 11204937) and China National Institute of Standardization (approval number: 5120218739).

#### 2.4.2. Data Analysis

The collected data were assessed for their reliability and validity to test the effectiveness of the scales. The internal consistency reliability was evaluated to test the consistency of multiple items for measuring the same construct [45]. Composite reliability was tested for each dimension to measure the reliability degree of compositional constructs [46]. Convergent validity was assessed by using composite reliability together with factor loading and average variance extracted (AVE) [47]. Discriminant validity was evaluated by comparing the square root of AVE with its largest inter-construct correlations for a certain factor [48]. Confirmatory factor analysis (CFA) was performed to evaluate the degree of fit of the measurement model to the research data. Goodness-of-fit indices (GFI), as well as root mean square residual (RMR), Tucker–Lewis index (TLI), root mean square error of approximation (RMSEA), chi-square divided by degree of freedom (x^2^/df), and comparative fit index (CFI), were used to determine whether the CFA model has a good fit to the data [49].

After the reliability and validity tests, descriptive analysis was conducted to present demographic distributions of the accumulated frequencies of reported SC and SCB scores for Hong Kong and Mainland China, and further compared for the significance of the between group differences through the analysis of variance (ANOVA) in terms of different demographic information [50]. Moreover, multinomial regression was used to predict the possible outcomes of SC and SCB with different categorizations of demographic information [51]. Structural equation modeling (SEM) was applied to quantitatively measure the influence mechanism of demographic factors in two regions. All the demographic variables were coded according to their categories. Table 2 presents the detailed coding system for the demographic variables.

## 3. Results

### 3.1. Validity and Reliability Test

The internal consistency reliability of the scale was tested by using Cronbach’s alpha, and Table 3 presents the results. The Cronbach’s alphas of SC and SCB for both Hong Kong and Mainland China exceeded 0.7, thereby indicating the high level of internal consistent reliability of the data [45]. Table 3 shows the good acceptances of the factor loadings (larger than 0.5) of all items for both regions. Table 4 shows that the composite reliabilities of SC and SCB for both regions exceeded 0.7, while the values of AVE were all larger than 0.5 [48]. Good convergent validity was therefore verified as reflected in the acceptability of composite reliabilities, values of AVE, and factor loadings [47,52,53].

Table 5 and Table 6 indicate that the largest Pearson correlation between different dimensions of SC and SCB is lower than the square root of AVE for each dimension. The discriminant validity of each dimension of SC and SCB was therefore verified [54].

As depicted in Table 7, the CFA model of Hong Kong was tested using the following criteria: χ2/df = 3.292, TLI = 0.954, CFI = 0.969, RMSEA = 0.021, GFI = 0.921, and RMR = 0.049, which altogether demonstrated a remarkable degree of fit of the measurement model to the questionnaire data. For Mainland China, a high degree of fit was also verified between the data and the model (χ2/df = 2.364, TLI = 0.967, CFI = 0.977, RMSEA = 0.044, GFI = 0.898, and RMR = 0.027) [49].

### 3.2. Descriptive Analysis

Table 8 shows the demographic distributions of the accumulated frequencies of reported SC and SCB scores for both Mainland China and Hong Kong. The highest proportion of respondents was observed to be 31–40 years old for two regions (63 from Mainland China, and 64 from Hong Kong). Most of the respondents had high school diplomas (71 from Mainland China, and 98 from Hong Kong) and worked 41 to 45 h per week (52 from Mainland China, and 58 from Hong Kong). Overall, male construction workers had higher SC and SCB scores than their female counterparts. Hong Kong construction workers showed higher SC and SCB scores than their Mainland counterparts.

**Figure 1:**
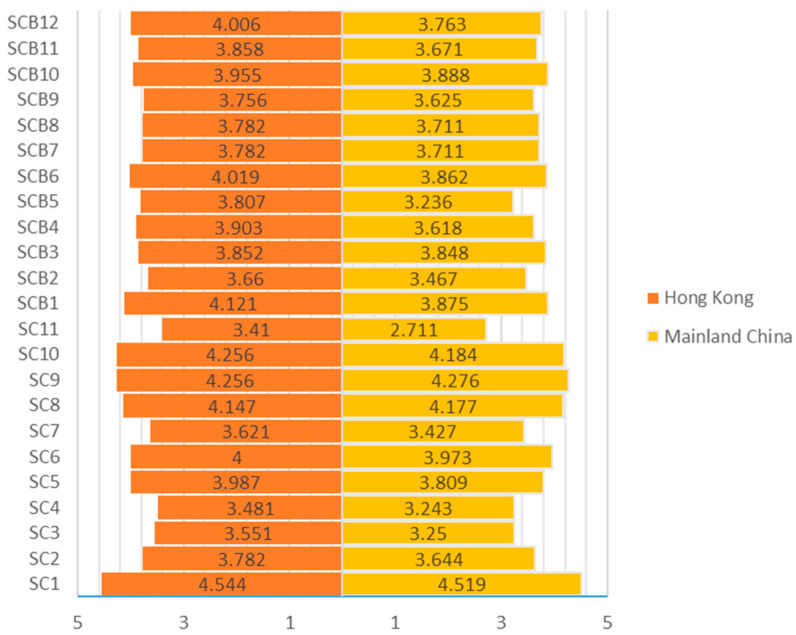
SC and SCB profiles of construction workers from Mainland China and Hong Kong.

The average scores of every item for SC and SCB were presented on a butterfly graph (Figure 1). Each questionnaire item was set as the axis, and the average score of each item was represented. As shown in Figure 1, the Hong Kong respondents only had two items lower than those of Mainland counterparts (SC8: “You think you should strengthen personal safety consciously during the construction process,” and SC9: “You are well aware of the terms of the building industry standards”).

### 3.3. ANOVA

For the comparison of the demographic differences in SC and SCB between Hong Kong and Mainland China, Table 9 and Table 10 depict the results of ANOVA to evaluate the significances of distinctions. Table 9 reveals significant differences in the SC and SCB of construction workers between regions (*p* < 0.05). As shown in Section 4.1, the SC and SCB of Hong Kong workers (3.974 and 3.891, respectively) were significantly higher than those of Mainland workers (3.756 and 3.718, respectively).

Table 10 reveals that all demographic variables significantly differentiate the SC and SCB of construction workers in Hong Kong and Mainland China, given that the values of *p* were all less than 0.001. Combined with the statistical analysis presented in Section 4.1, the SC and SCB of male workers were significantly higher than those of female workers in two regions. SC and SCB of workers with higher education level were significantly higher than those with lower education level in both regions. Workers with longer working hours reported significantly lower SC and SCB in two regions. Older construction workers had significantly higher SC and SCB than younger workers in Mainland China, whereas the SC and SCB decreased along with increasing age in Hong Kong.

### 3.4. Multinomial Regression

Table 11 and Table 12 present the multinomial regression results for SC and SCB of construction workers in Mainland China and Hong Kong, respectively. All coefficients of the demographic variables were verified as significant in these two regions, given that the values of *p* were all less than 0.001. No multiple collinearity was observed among the different factors in accordance with the acceptable tolerance and variance inflation factor (VIF). The regression constants refer to the intercept of the function. For Hong Kong, the regression constants for SC and SCB were 3.525 and 4.588, respectively, indicating greater intercepts compared with those of Mainland China (2.226 for SC and 3.360 for SCB). The values of adjusted R^2^ represent the degrees of explanation of the variables toward the variations of SC and SCB.

### 3.5. Demographic Influence Modeling

**Figure 2:**
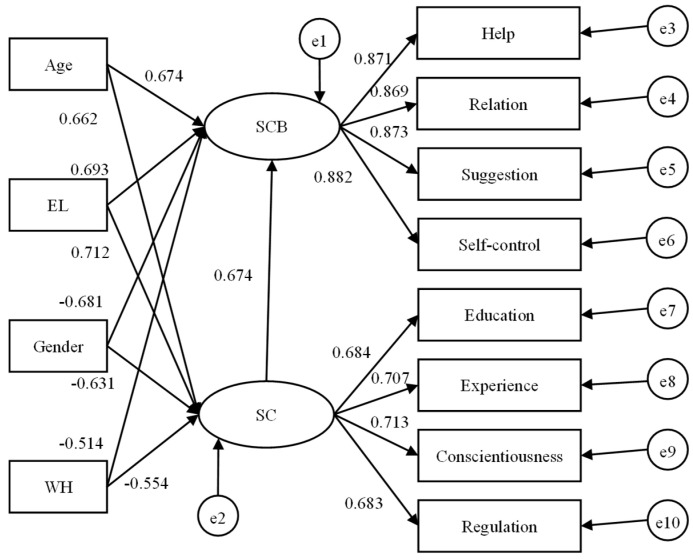
Structural equation model of the demographic influence on the SC and SCB in MC.

**Figure 3:**
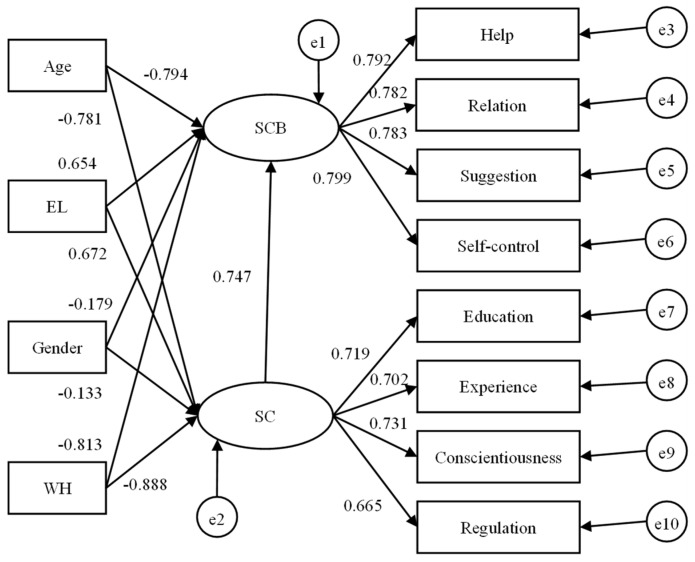
Structural equation model of the demographic influence on the SC and SCB in HK.

To compare the influence mechanism of demographic variables on the SC and SCB of construction workers in Hong Kong and Mainland China quantitatively, structural equation modeling (SEM) was performed to estimate the impact of different demographic variables on SC and SCB, as shown in Figure 2 and Figure 3. Table 13 depicts that both SC and SCB models exhibited good fit to the data. As is vividly shown in the figures, the negative effects in the Mainland China model were verified in line with the coefficients of gender (coded as 0 for male and 1 for female) and weekly working hours (WH). Meanwhile, age and education level (EL) showed positive effects on SC and SCB. For Hong Kong, only EL showed a positive effect on SC and SCB, whereas gender, age, and WH showed negative effects to both safety constructs according to their coefficients.

In order to compare the demographic influence on SC and SCB between Hong Kong and Mainland China, test of invariance routine was conducted to identify performing distinction of the research model between regions [55]. The differences of goodness-of-fit indices were examined and listed in Table 14, which reveals significant distinction of SEM between two regions in terms of demographic influence on SC and SCB [55].

Furthermore, the significances of different influence paths were verified as shown in Table 15, in which all paths were significant given that the *p* values were all less than 0.05, though the strength of the demographic effect was partially different between two regions. Specifically, a stronger effect of gender was shown in Mainland China, while the stronger influences of workhour and age were found in Hong Kong.

Therefore, H1 was verified because the SC and SCB of Mainland workers were lower than those of Hong Kong workers. H2, H2.1 and H2.3 were all accepted since the significant negative influence of gender was greater in Mainland China (0.005 significance level) compared with that in Hong Kong (0.05 significance level), which further led to the rejection of H2.2. H3 and H3.1 were supported but H3.2 and H3.3 were rejected given the positive effect of education on SC and SCB in two regions, which were all significant at 0.005 level. H4 was accepted due to the significance of age effect but H4.1, H4.2 and H4.3 were all rejected given the negative effect of age in Hong Kong and positive effect in Mainland China. H5, H5.1 and H5.2 were supported given the stronger negative effects of weekly working hours in Hong Kong at a 0.001 significance level, which further led to the rejection of H5.3.

## 4. Discussion

### 4.1. Regional Similarity

#### 4.1.1. Territorial Education Strategies

Workers with insufficient education have a limited understanding of safety and working capabilities [56]. Male and female workers in both regions with higher education were reported to have higher SC and SCB scores. The influence of education on SC and SCB were significant at a 0.005 level in both Hong Kong and Mainland China, which validated the effectiveness of education in enhancing the SC and SCB of workers. Generally, the education level of Mainland workers is lower than Hong Kong workers because more of the latter obtained high school degree (98 respondents), and more of the former only obtained junior middle school degree or below (58 respondents). The relevant Mainland authorities should be concerned that the lower education level of the workers will create a huge obstacle toward the understanding of the safety education because of the shortages of general knowledge and learning capacities [57]. Therefore, using graphical presentation and example illustration is recommended in areas that are difficult to understand while conducting on-job campaigns and safety courses, preferably with rewards and incentives for workers to ensure the learning initiative, such as safety performance reward or paid learning time [58]. However, in this study, Hong Kong respondents reported lower scores in terms of the safety regulation of construction industry (Item: SC9) than their Mainland counterparts. Therefore, workers education in Hong Kong should be more emphasized on the learning of the construction regulation to facilitate their proper understanding and familiarity to the common ordinance and laws of Hong Kong construction industry, such as “Fire Safety (Building) Ordinance, CAP 572” and “Building Ordinance, CAP 123”.

#### 4.1.2. Workhour Design

Workhour was identified in both regions as a significant predictor of occupational injuries, with more workload corresponding to lower SC and SCB. One possible explanation for such finding is that a prolonged working time will exhaust workers and reduce their concentration and consciousness, thereby negatively affecting their attitudes and organizational participations toward work safety [36,59]. To solve this problem, working duration should be properly scheduled to optimize the working efficiency and safety performance of construction workers [10]. Rest intervals should be integrated to guarantee the recovery of worker’s physical strength. Additionally, adverse weather conditions should be considered in the design of workhours as a stressor of workload [60]. To illustrate, outdoor works, such as masonry and earth excavation, starting from 12 pm during midsummer should be suspended and delayed until 3 pm due to the physical consumption and work burnout caused by high temperature.

### 4.2. Regional Differentia

#### 4.2.1. Individual and Organizational Motivation

SC of the respondents from Hong Kong is higher than that of the respondents from Mainland China, which may be attributed to the carrying out of safety incentive systems and high levels of professional education in the Hong Kong construction industry. The Hong Kong government has introduced a “Pay for Safety Scheme” project, where contractors planning to tender for public infrastructural works can include several safety-related tasks as part of their bills of quantities. These contractors will be paid for these items when these tasks are successfully implemented and achieved, which can increase the conscientiousness and motivation of managers and workers [61]. Moreover, the Hong Kong Occupational Safety and Health Council has organized training courses since 1988 for construction personnel who aimed to promote site safety [62]. Therefore, the construction management of Mainland China is suggested to take Hong Kong as an example by conducting safety incentive system and site safety education for workers to enhance both conscientiousness and safety knowledge, thereby further improving SC [8].

Construction workers in Hong Kong were verified to have better SCB than their counterparts in Mainland China because of the high education level (more Hong Kong respondents obtained a high school degree). The management and supervisory staff groups in the Hong Kong are highly educated and concerned about the safety of their coworkers and employees [63]. Management believe that the poor safety of personnel will negatively affect the reputations of the company and induce high compensation. Therefore, they tend to play a proactive role to guarantee the safety of their workers and protect the interests of their organizations [64,65]. By contrast, the managers of construction companies in Mainland China lack authority to make decisions and are not responsible for the profits and losses of the projects. Therefore, they generally lack the motivation to carry out their work with cost-effectiveness [66,67]. The present study recommends that the relevant authorities and management of Mainland China conduct a reward mechanism for safety cooperation of construction employees, such as remuneration and extra vacation for personnel who make prominent contributions to organizational safety [68], as well as the continuing education of high-quality leadership for construction management and group leaders to improve the SCB of construction workers by learning from the experience of Hong Kong [69].

#### 4.2.2. Ageing Problem

To compare the difference in different age groups, the workers below 20 years old in Hong Kong performed better SC and SCB than those in Mainland China, which is mainly due to the superior education popularization and quality in Hong Kong. With the age increasing, the changing trends of SC and SCB were shown to move in the opposite direction. The elder workers in Mainland China reported higher SC and SCB, which perceive more support and encouragement from organization and are more willing to wear the safety equipment than their younger counterparts [70]. These elder workers also realize that few job opportunities are available to them, thereby driving them to show higher commitment to their work and more willingness to obey safety regulations of the supervisor [71,72]. By contrast, the negative relationship of age with SC and SCB in the construction industry of Hong Kong was clarified, which is mostly attributed to the declining trajectories of the working ability and retirement pathways of the Hong Kong workers [73]. Specifically, Peng and Chan considered reduced working capacity and psychological engagement as the obstacles to personnel safety among the elder workers in Hong Kong [74]. They further attributed the reduced risk perception and avoidance of Hong Kong elder workers to the changes in the trajectories of working ability along with age [75], which negatively affect the conscientiousness of risk avoidance and working cooperation [76,77].

The study further analyzed the slope coefficients (absolute value) of ageing effect in two regions, which revealed the influential strength (positive or negative) of ageing in different periods of time. As shown in Table 16, the effect of ageing was proven to be stronger on SC and SCB between groups with age codes 1 and 2. The effect continued to decline before the age of 45–50, after which the impact of ageing was recovered to be predominate after the individual is more than 50 years old. The findings can be corroborated by the non-linear development and senility of personnel’s body function and psychological cognition [78].

In addition, the findings reveal that additional workhours will cause a stronger negative effect on SC and SCB of Hong Kong workers than Mainland workers, which is mainly due to the large proportion of ageing workforce of the Hong Kong construction industry, with insufficient physical and psychological strength to achieve consciousness concentrating and provide altruistic assistance under the excessive workload [2,79].

Therefore, the relevant authorities and managements of the Hong Kong construction companies are advised to improve the physical capacity of elder workers by organizing trans-theoretical model-based educational programs involving different activities, such as lectures, training workshops, group discussions, and propaganda regarding regular physical activity [80]. Moreover, concerned authorities could hold safety promotional campaigns to increase the mental health of elder workers, preferably involving control interventions, job stress prevention, help-seeking promotion, mental health literacy improvement, and the establishment of positive leadership practices [81].

#### 4.2.3. Female Caring

The SC and SCB of female workers are generally lower than their male counterparts. Female workers in Mainland China performed worse SC and SCB under a stronger gender effect than their Hong Kong counterparts, which mainly attributed the poor SC and SCB of Mainland female workers of their working marginalization due to the hegemonic masculinity of males, which is specifically generated from the traditional culture in Mainland China and not predominant in Hong Kong due to the difference of regional culture and social background [82]. Therefore, the intensity of gender effect in Hong Kong is relatively moderate. The work schedule and condition should be specially designed for female workers with the consideration of ensuring their occupational health, especially for Mainland China [83]. The heavy jobs, such as manual handling and reinforcing works, should be reduced for female workers due to their low physical capacity, and additional rest interval should be properly scheduled [84]. Furthermore, the perception of organizational affiliation should be promoted for female workers by increasing the support and commitment from all levels of supervision and management through practical measures, such as the Given Voice to Value approach, so as to help them achieve high cohesion and unity with group members [85].

## 5. Conclusions

### 5.1. Theoretical and Practical Contributions

This study provides insights into the comparison of demographic influence on SC and SCB of construction workers between Hong Kong and Mainland China. As one of its important theoretical contributions, this study firstly recommends a specific focus on the territorial comparison of SC and SCB to design targeted and effective measures and suggestions for SC and SCB improvement. Specifically, the gender and workhour caused negative effect towards SC and SCB in both regions, while the effect of education was positive. SC and SCB of Hong Kong workers were higher than those of Mainland workers. Although the influence of age was shown to be negative in Hong Kong, the positive impact of age was verified in Mainland China.

The insights generated by this study offer construction management practical implications. The findings put forward a territorial plan of safety education for construction workers in Hong Kong and Mainland China, respectively. Moreover, the optimized workload and work conditions are discussed with consideration of environmental and gender characteristics in both regions. In addition, the construction management is recommended to carry out safety incentive and reward mechanisms in Mainland China for cooperation and contribution of construction employees, while the Hong Kong construction companies should improve the work safety of elder workers by organizing trans-theoretical model-based educational programs.

### 5.2. Limitations

This research still has certain limitations. First, the scope of demographic information can be further expanded, and the length of working service can be added as additional demographic information to enhance the effectiveness of the research. Second, the data obtained from the survey were cross-sectional, and the tested effects of demographic variables only focused on a static time point instead of the overall process, which may partially eliminate the interpretation of the influence mechanism toward SC and SCB. Future research is suggested to improve the data collection process by distributing and collecting data over multiple periods of time.

## Figures and Tables

**Table 1:** Hypotheses for demographic impact on SC and SCB of construction workers.

No.	Content
H1	Hong Kong construction workers perform higher SC and SCB than Mainland construction workers.
H2	Gender difference causes significant influence on the SC and SCB of construction workers in two regions.
H2.1	Gender difference causes negative influence on the SC and SCB of construction workers in two regions.
H2.2	Gender causes stronger negative effect towards SC and SCB of Hong Kong workers.
H2.3	Gender causes stronger negative effect towards SC and SCB of workers from Mainland China.
H3	Education level causes significant influence on the SC and SCB of construction workers in two regions.
H3.1	Education level causes positive influence on the SC and SCB of construction workers in two regions.
H3.2	Education level causes stronger positive effect towards SC and SCB of Hong Kong workers.
H3.3	Education level causes stronger positive effect towards SC and SCB of workers from Mainland China.
H4	Age causes significant influence on the SC and SCB of construction workers in two regions.
H4.1	Age causes positive influence on the SC and SCB of construction workers in two regions.
H4.2	Age causes stronger positive effect towards SC and SCB of Hong Kong workers.
H4.3	Age causes stronger positive effect towards SC and SCB of workers from Mainland China.
H5	Working hour causes significant influence on the SC and SCB of construction workers in two regions.
H5.1	Working hour causes negative influence on the SC and SCB of construction workers in two regions.
H5.2	Working hour causes stronger negative effect towards SC and SCB of Hong Kong workers.
H5.3	Working hour causes stronger negative effect towards SC and SCB of workers from Mainland China.

**Table 2:** Coding system for the demographic variables of the questionnaire.

Age	Gender	Education Level	Weekly Working Hours
[<20]—1	Male—0	Junior middle school or below—1	[<35]—1
[20–30]—2	Female—1	High school—2	[35–40]—2
[31–40]—3		Technical school—3	[41–45]—3
[41–50]—4		Undergraduate or above—4	[46–50]—4
[>50]—5			[51–55]—5
			[>55]—6

**Table 3:** Results of factor loadings and Cronbach’s alphas.

Safety Construct	Dimension	Item	Factor Loading (HK)	Factor Loading (MC)	Cronbach’s Alpha (HK)	Cronbach’s Alpha (MC)
Safety consciousness	Education	Item 1	0.756	0.702	0.801	0.800
		Item 2	0.859	0.908		
		Item 3	0.854	0.825		
	Experience	Item 4	0.819	0.831		
		Item 5	0.981	0.892		
		Item 6	0.895	0.910		
	Conscientiousness	Item 7	0.937	0.880		
		Item 8	0.921	0.924		
	Regulation	Item 9	0.873	0.884		
		Item 10	0.888	0.788		
		Item 11	0.922	0.808		
Safety citizenship behavior	Mutual help	Item 1	0.987	0.803	0.883	0.921
		Item 2	0.973	0.851		
		Item 3	0.913	0.883		
	Relation exchange	Item 4	0.807	0.929		
		Item 5	0.813	0.571		
		Item 6	0.892	0.843		
	Suggestion	Item 7	0.957	0.862		
		Item 8	0.916	0.845		
		Item 9	0.894	0.866		
	Self-control	Item 10	0.729	0.926		
		Item 11	0.906	0.933		
		Item 12	0.872	0.903		

**Table 4:** Results of composite reliability and average variance.

Dimension	Composite Reliability (HK)	Average Variance Extracted (HK)	Composite Reliability (MC)	Average Variance Extracted (MC)
Education	0.863	0.679	0.855	0.666
Experience	0.927	0.811	0.909	0.771
Conscientiousness	0.926	0.863	0.897	0.814
Regulation	0.923	0.800	0.866	0.685
Mutual help	0.971	0.918	0.883	0.716
Relation exchange	0.876	0.702	0.833	0.633
Suggestion	0.944	0.851	0.893	0.735
Self-control	0.876	0.704	0.943	0.847

**Table 5:** Inter-factor confirmatory correlations among the latent variables for Hong Kong.

	Education	Experience	Conscientiousness	Regulation	Mutual Help	Relation Exchange	Suggestion	Self-Control
Education	0.824							
Experience	0.486 **	0.901						
Conscientiousness	0.596 **	0.538 **	0.929					
Regulation	0.449 **	0.318 **	0.631 **	0.894				
Mutual help	0.643 **	0.689 **	0.734 **	0.600 **	0.958			
Relation exchange	0.708 **	0.587 **	0.733 **	0.658 **	0.645 **	0.838		
Suggestion	0.717 **	0.535 **	0.742 **	0.673 **	0.621 **	0.652 **	0.922	
Self-control	0.674 **	0.678 **	0.750 **	0.601 **	0.646 **	0.647 **	0.616 **	0.839

The diagonal values refer to the square roots of AVE. **: Significant correlation at the 0.01 level (two-tailed).

**Table 6:** Inter-factor confirmatory correlations among the latent variables for Mainland China.

	Education	Experience	Conscientiousness	Regulation	Mutual Help	Relation Exchange	Suggestion	Self-Control
Education	0.816							
Experience	0.497 **	0.878						
Conscientiousness	0.586 **	0.545 **	0.902					
Regulation	0.370 **	0.266 **	0.619 **	0.828				
Mutual help	0.699 **	0.696 **	0.823 **	0.618 **	0.846			
Relation exchange	0.693 **	0.639 **	0.768 **	0.669 **	0.723 **	0.796		
Suggestion	0.681 **	0.683 **	0.768 **	0.633 **	0.781 **	0.700 **	0.857	
Self-control	0.705 **	0.707 **	0.813 **	0.633 **	0.806 **	0.790 **	0.739 **	0.920

The diagonal values refer to the square roots of AVE. **: Significant correlation at the 0.01 level (two-tailed).

**Table 7:** Criteria of CFA results for Hong Kong and Mainland China.

Questionnaire	$\chi^{2}/df$	RMR	GFI	TLI	CFI	RMSEA
Hong Kong	3.292	0.049	0.921	0.954	0.969	0.021
Mainland	2.364	0.027	0.898	0.967	0.977	0.044
Criterion	<5	<0.05	>0.9	>0.9	>0.9	<0.05

**Table 8:** Statistical analysis of the SC and SCB performances of construction workers.

Demographic		Mainland China (253)					Hong Kong (256)				
		N	Average SC	Average SCB	SD (SC)	SD (SCB)	N	Average SC	Average SCB	SD (SC)	SD (SCB)
Gender	Male	141	3.981	4.001	0.355	0.411	142	4.263	4.187	0.247	0.326
	Female	112	3.472	3.362	0.533	0.583	114	3.613	3.523	0.344	0.387
Education level	Junior middle school or below	58	2.951	3.042	0.317	0.433	42	3.175	3.265	0.324	0.437
	High school	71	3.657	3.604	0.287	0.324	98	3.619	3.515	0.279	0.343
	Technical school	69	4.099	3.951	0.197	0.216	63	4.242	4.253	0.165	0.221
	Undergraduate or above	55	4.299	4.291	0.277	0.244	53	4.740	4.431	0.166	0.163
Age	<20	41	3.301	3.383	0.175	0.127	47	4.451	4.441	0.186	0.177
	20–30	55	3.421	3.499	0.144	0.186	53	4.296	4.315	0.193	0.216
	31–40	63	3.681	3.658	0.177	0.218	64	3.858	3.721	0.279	0.278
	41–50	50	4.156	3.951	0.262	0.215	57	3.578	3.411	0.398	0.349
	>50	44	4.257	4.123	0.390	0.325	35	3.361	3.272	0.410	0.395
Weekly working hours	<35	37	4.391	4.358	0.180	0.249	28	4.428	4.461	0.197	0.174
	36–40	41	4.051	4.142	0.186	0.233	42	4.233	4.204	0.154	0.251
	41–45	52	3.734	3.761	0.212	0.282	58	4.159	4.062	0.257	0.238
	46–50	49	3.615	3.593	0.240	0.332	55	3.834	3.782	0.368	0.385
	51–55	37	3.442	3.378	0.333	0.413	40	3.532	3.404	0.433	0.388
	>55	37	3.328	3.055	0.325	0.419	33	3.341	3.123	0.435	0.432
Total average			3.756	3.718	0.669	0.802		3.974	3.891	0.674	0.821

**Table 9:** ANOVA results for SC and SCB in Hong Kong and Mainland China.

			Quadratic Sum	Df	Mean Square	F	*p* (Sig.)
Region	SC	Interclass	1.311	1	1.311	5.685	0.01 **
		Intraclass	116.899	507	0.231		
		Total	118.210	508			
	SCB	Interclass	1.655	1	1.654	5.267	0.01 **
		Intraclass	159.304	507	0.314		
		Total	160.959	508			

**: *p* < 0.01, which indicates significance of between group difference.

**Table 10:** Comparative ANOVA results for the SC and SCB of construction workers.

Feature	Constructs	Sig of HK (*p*)	Sig of MC (*p*)
Gender	SC	0.000 ****	0.000 ****
	SCB	0.000 ****	0.000 ****
Age	SC	0.000 ****	0.000 ****
	SCB	0.000 ****	0.000 ****
Education level	SC	0.000 ****	0.000 ****
	SCB	0.000 ****	0.000 ****
Weekly working hours	SC	0.000 ****	0.000 ****
	SCB	0.000 ****	0.000 ****

****: *p* < 0.001, which indicates significance of between group difference.

**Table 11:** Multinomial regression of the SC and SCB of construction workers in Hong Kong.

Regression Models		Unstandardized Coefficients		Standardized Coefficients	t	*p* (Sig.)	Collinearity Statistics		Adjusted R^2^
		B	Std. Error	Beta			Tolerance	VIF	
SC	(Constant)	3.525	0.154		29.341	0.000			0.684
	Gender	−0.336	0.066	−0.244	−4.127	0.000	0.698	1.433	
	Age	−0.324	0.038	−0.415	−6.740	0.000	0.642	1.557	
	Education level	0.163	0.028	0.328	5.839	0.000	0.774	1.293	
	Working hours	−0.149	0.027	−0.268	5.570	0.000	0.879	1.138	
SCB	(Constant)	4.588	0.159		28.785	0.000			0.749
	Gender	−0.321	0.068	−0.257	−4.726	0.000	0.643	1.556	
	Age	−0.294	0.039	−0.429	−7.574	0.000	0.752	1.330	
	Education level	0.194	0.029	0.347	6.736	0.000	0.761	1.314	
	Working hours	−0.174	0.027	−0.279	−6.499	0.000	0.899	1.112	

Note: VIF: Variance Inflation Factor, which should be less than 10 to indicate the absence of collinearity.

**Table 12:** Multinomial regression of the SC and SCB of construction workers in Mainland China.

Regression Models		Unstandardized Coefficients		Standardized Coefficients	T	*p* (Sig.)	Collinearity Statistics		Adjusted R^2^
		B	Std. Error	Beta			Tolerance	VIF	
SC	(Constant)	2.226	0.060		37.327	0.000			0.941
	Age	0.271	0.033	0.492	8.126	0.000	0.926	1.080	
	Education	0.108	0.039	0.161	2.778	0.006	0.278	3.592	
	Gender	−0.052	0.026	−0.037	−2.000	0.047	0.868	1.152	
	Working hours	−0.145	0.037	−0.328	−3.944	0.000	0.289	3.456	
SCB	(Constant)	3.360	0.371		9.063	0.000			0.948
	Age	0.286	0.045	0.450	6.435	0.000	0.912	1.096	
	Education	0.119	0.052	0.153	2.295	0.023	0.322	3.106	
	Gender	−0.122	0.035	−0.076	−3.520	0.001	0.794	1.259	
	Working hours	−0.181	0.049	−0.354	−3.690	0.000	0.381	2.625	

Note: VIF: Variance Inflation Factor, which should be less than 10 to indicate the absence of collinearity.

**Table 13:** Model fit indices for the demographic influence of the SEMs.

	$\chi^{2}/df$	SRMR	TLI	CFI	RMSEA	GFI	AGFI	PGFI
Hong Kong model	2.139	0.026	0.978	0.988	0.057	0.912	0.837	0.591
Mainland China model	2.966	0.042	0.965	0.977	0.065	0.903	0.924	0.598
Standard	≤5	≤0.08	≥0.9	≥0.9	≤0.08	≥0.5	≥0.8	≥0.5

**Table 14:** Comparisons for cross-regional structural equation models.

Comparison	$∆SB-\chi^{2}$	∆df	∆NNFI	∆CFI
Hong Kong vs. Mainland China	66.16 **	19 **	0.010 **	0.011 **

Note. SB-χ2 = Sattora–Bentler scaled chi-square; df = model degrees of freedom; CFI = comparative fit index; NNFI = non-normed fit index; RMSEA = root-mean squared error of approximation. **: *p* < 0.01.

**Table 15:** Significance of influence paths among the demographic variables and safety constructs.

Path			Path Coefficient (HK)	Sig (HK)	Path Coefficient (MC)	Sig (MC)
SC	<---	EL	0.672	***	0.712	***
SC	<---	Gender	−0.133	*	−0.631	***
SC	<---	WH	−0.888	****	−0.554	***
SC	<---	Age	−0.781	****	0.662	***
SCB	<---	Age	−0.794	****	0.674	***
SCB	<---	EL	0.654	***	0.693	***
SCB	<---	Gender	−0.179	**	−0.681	***
SCB	<---	WH	−0.813	****	−0.514	***

*: *p* < 0.05, **: *p* < 0.01, ***: *p* < 0.005, ****: *p* < 0.001, WH: weekly working hour, EL: education level.

**Table 16:** Slope coefficient of ageing effect towards SC and SCB in Hong Kong and Mainland China.

Codes of Age	Slope Coefficient (Absolute Value)							
	Mainland China				Hong Kong			
	SC		SCB		SC		SCB	
1–2	8.331	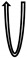	8.622	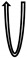	6.455	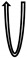	7.948	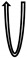
2–3	3.852		6.291		2.287		1.689	
3–4	2.113		3.417		3.572		3.233	
4–5	9.904		5.816		4.611		7.192	

Note: for age codes, “<20”—1; “20–30”—2; “31–40”—3; “41–50”—4; “>50”—5.

## Data Availability

The data presented in this study are available on request from the corresponding author. The data are not publicly available due to privacy and ethical considerations.
